# A dual-detector optical receiver for PDM signals detection

**DOI:** 10.1038/srep26469

**Published:** 2016-05-20

**Authors:** Guanyu Chen, Yu Yu, Xinliang Zhang

**Affiliations:** 1 Wuhan National Laboratory for Optoelectronics & School of Optical and Electronic Information, Huazhong University of Science and Technology, Wuhan 430074, China

## Abstract

We propose and fabricate a silicon based dual-detector optical receiver, which consists of a two dimensional (2D) grating coupler (GC) and two separate germanium photodetectors (Ge PDs). The 2D GC performs polarization diversity, and thus demultiplexing and detection for polarization division multiplexed (PDM) signals can be achieved. Through a specific design with double-sides illumination, the space charge density can be reduced and the responsivity and saturation power can be improved significantly. The measured dark current, responsivity and bandwidth are 0.86 μA, 1.06 A/W and 36 GHz under 3 V reverse biased voltage, respectively. Both DC currents and eye diagrams are measured for the proposed device and the results validate its performance successfully. The power penalty between the single and dual polarized signals is about 1.9 dB under 10 and 20 Gb/s cases for both the two Ge PDs. The proposed direct detection (DD) for PDM signals with high speed, high responsivity and large saturation power is cost-effective and promising for short reach optical communication.

To meet the increasing demand for large optical communication capacity, multidimensional multiplexing technology is adopted both in fibers and integrated chips^1^,^2^,^3^,^4^,^5^,^6^,^7^,^8^,^9^. Among various multiplexing technologies, the polarization division multiplexing (PDM) has received a lot of interests as it doubles the transmission capacity^10^. Due to the low cost and easy compatibility with complementary metal oxide semiconductor (CMOS) technology, silicon on insulator (SOI) platform has attracted great attention^11^,^12^,^13^ and SOI based multidimensional multiplexing were widely studied in particular^3^,^4^,^5^,^6^,^7^,^8^,^9^. The polarization multiplexing/demultiplexing has been successfully realized on silicon^3^,^4^,^6^ and many silicon based devices for PDM signal processing are proposed^14^,^15^,^16^,^17^,^18^,^19^,^20^,^21^. As one of the key components in silicon based photonic integrated circuits, an optical receiver suitable for both PDM signal detection and demultiplexing is greatly needed. Although the dual polarization coherent receiver had been reported utilizing either the two dimensional (2D) grating coupler (GC)^17^,^18^ or the polarization handling elements^20^,^21^, the direct detection (DD) with simple structure and low cost is on the other hand promising for some specific applications such as the short reach link^22^,^23^,^24^,^25^,^26^,^27^,^28^,^29^, where the coherent detection is not necessary. Most of the reported schemes were based on discrete photodetectors (PDs) combined with polarization handling devices. Taking the advantage of integrated photonic devices, the same function can be realized on-chip and the complexity of the receiver will thus be reduced greatly.

In this paper, we therefore propose and fabricate a dual-detector optical receiver suitable for PDM signals direct detection. A 2D GC is adopted to perform the polarization diversity, and two separate germanium (Ge) PDs are utilized to detect the two polarization tributes. The four ports of the 2D GC are fully used and each two opposite ports connect to two sides of the Ge absorption regions. Taking advantage of the double-sides illumination, the space charge density can be reduced and high responsivity and large saturation power can be obtained. The coplanar waveguide electrodes connected to the Ge regions are used for the electrical signals transmission. The dark current, responsivity and bandwidth are measured to be 0.86 μA, 1.06 A/W and 36 GHz under 3 V reverse biased voltage, respectively. The DC currents and eye diagrams are measured from the two PDs when the input signals are polarization multiplexed and the results show the successful detection and demultiplexing of PDM non-return-to zero (NRZ) signals. For quantitative analysis of the proposed dual-detector optical receiver, the bit error ratio (BER) measurements are carried out, and the power penalty is about 1.5 dB between the single and dual polarization states for the two Ge PDs.

## Results

### Operation principle and design

**Figure 1:**
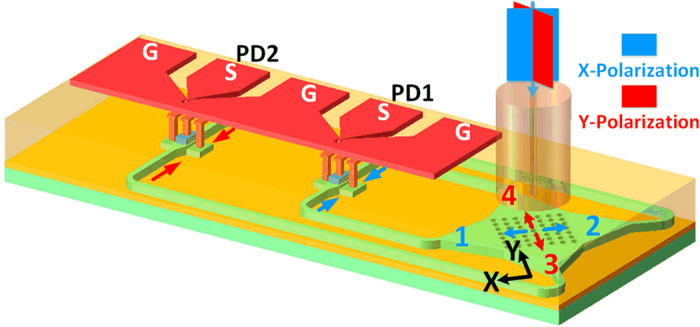
Schematic diagram. The schematic diagram of the proposed optical receiver (dimensions are not drawn to scale).

The schematic diagram of the proposed device is shown in Fig. 1. Such an optical receiver consists of a 2D GC and two separate Ge absorption regions (PD1 and PD2) sharing a common ground-signal-ground-signal-ground (GSGSG) lumped electrode. The transmitted PDM-NRZ signals (with X and Y polarization states) in the fibers are coupled into the silicon waveguide through the GC. The four ports (denoted as 1, 2, 3 and 4) of the 2D GC are fully used and each two opposite ports connect to two sides of the Ge absorption region through the silicon waveguide.

If the input signal with X polarization state is aligned to the X direction of the 2D GC, it will be coupled into the two waveguides connected to the two sides of the PD1 through ports 1 and 2. Meanwhile, the input signal with Y polarization aligned to the Y direction will transmit to the PD2 through ports 3 and 4. Therefore, the demultiplexing and detection of the PDM signals can be realized at the same time based on the proposed receiver. As the coupled signals in the waveguide through the 2D grating are on the same polarization state (TE), the waveguide and Ge absorption regions do not need special design. In order to ensure the same transmission for X and Y polarizations, the optical paths to PD1 and PD2 are designed with same length.

The 2D grating coupler is designed for vertical coupling. It is a square array of holes with an etch depth of 130 nm and a diameter of 340 nm. The lattice period is 590 nm and the total size of the 2D grating coupler is 13 μm × 13 μm.

**Figure 2:**
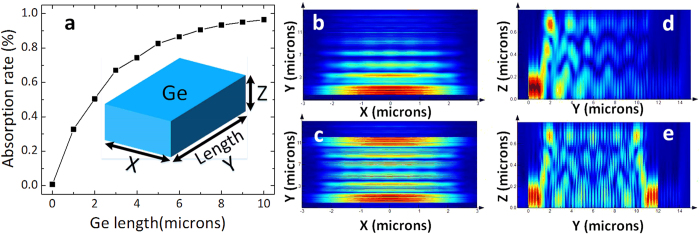
Simulation results. (**a**) The simulated Ge absorption rate vs. Ge length for single side illuminated case (the inserted figure is the 3D schematic diagram of Ge region). (**b**) The simulated optical field distribution at the XY cross section of the Ge region for Ge PDs with single-side and (**c**) double-sides illumination. (**d**) The simulated optical field distribution at the YZ cross section of the Ge region for Ge PDs with single-side and (**e**) double-sides illumination.

It should be noted that the responsivity and power handling capacity of the proposed device is improved because the Ge absorption regions are double-sides illuminated. There are two main advantages for double-sides illumination. One is more complete absorption of the input light under a fixed Ge length, and the other is more uniform optical field distribution. Figure 2(a) shows the simulated Ge absorption rate related to the Ge length for the single side illuminated TE mode signal at 1550 nm. It can be observed that not all the input light can be absorbed completely when the Ge length is 10 μm and about 4% input light will be lost due to the incomplete absorption. Of course the Ge length can be increased to realize more complete absorption, the bandwidth of the PD will however degrade consequently due to the large capacitance induced by the longer Ge region. If the absorption region is double-sides illuminated, a more thorough absorption will be obtained because the whole region is effectively used. Figure 2(b–e) show the simulated optical field distribution at the XY and YZ cross section of the Ge absorption region for single-side and double-sides illumination. A more uniform optical field distribution can be observed for the double-sides illumination case after comparing Fig. 2(b–e). As the space charge density will be reduced for the uniform optical field distribution case, the power handling capacity is improved for the structure with double-sides illumination.

**Figure 3:**
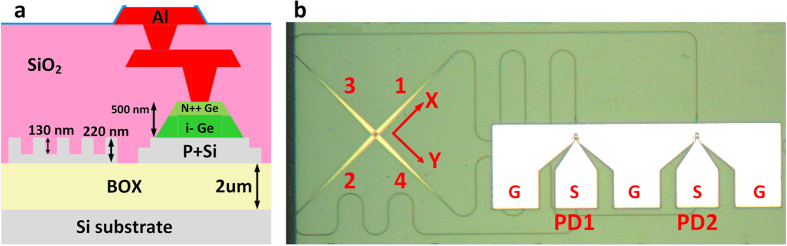
Cross section and microscopic image of the proposed device. (**a**) Cross section of the devices (dimensions are not drawn to scale). (**b**) Microscopic image of the fabricated receiver.

The proposed device is fabricated on the 220 nm thick SOI wafer with 2 μm buried oxide (BOX) using 0.18 μm CMOS technology at the Institute of Microelectronics (IME) in Singapore. Fully etched channel waveguide with 500 nm width is designed for single mode operation. The width and length of the Ge absorption region are designed to be 5 and 10 μm, receptively. A 500 nm thick germanium is grown on the P+ doping region of the silicon based on the two-step Ge growth procedure, and about 100 nm N++ doping region is on top of germanium to form the vertical PIN junction and N type ohmic contact. The double-layer metals are then deposited on the heavy doping area to form the electrode. The cross section of the devices is shown in Fig. 3(a) and the microscopic image of the fabricated receiver is shown in Fig. 3(b).

### DC current measurement

**Figure 4:**
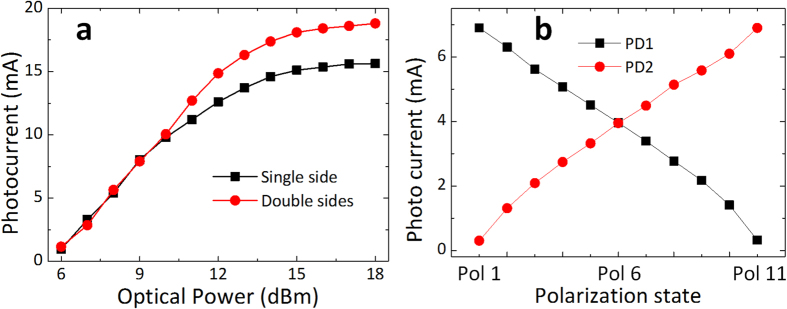
The DC current measurement results. (**a**) The measured photo currents as a function of input optical power for Ge PDs with single-side and double-sides (one PD of the dual-detector device) illuminated under 3 V reverse biased voltage. (**b**) The measured photo currents for the two separate Ge PDs when the input signals on different polarization states (Pol 1, Pol 6 and Pol 11 represent the X, 45° linear and Y polarization, respectively).

The DC characteristics are firstly measured for the proposed device. The dark current is measured to be 0.86 μA under 3 V reverse biased voltage. To verify its power handling capacity, the photo currents under different input optical power are measured for the Ge PDs with single-side and double-sides (one PD of the dual-detector device is measured for demonstration) illuminated and the results are shown in Fig. 4(a). The single-side case is measured using a reference PD with only one side illuminated. The measured maximum photo currents are 15.6 and 18.8 mA for the single-side and double-sides illuminated cases. The coupling loss of the 2D grating coupler is measured to be ~6 dB based on a reference structure. The 3 dB bandwidth and the polarization extinction ratio (PER) are also measured to be 40 nm and 14.5 dB, respectively. In the linearity region, the responsivities are calculated to be 1 and 1.06 A/W for the Ge PDs with single-side and double-sides illuminated under 3 V reverse biased voltage, respectively. The improved responsivity and power handling capacity can thus be validated.

Then the DC currents from the two PDs when the input CW light at 1550 nm under different polarization states are measured. The polarization states of the input signal is adjusted through changing the polarization controller (PC), and the two photo currents are collected through the GSGSG probe simultaneously. Meanwhile, the reverse biased voltage is applied through the bias-tee. The measured results are shown in Fig. 4(b). Results indicate that when the X polarization state (Pol 1 in Fig. 4) of the input light is align to the X direction of the 2D GC, most of the input light will be coupled into port1 and port2, and thus PD1 shows the large photo current compared with PD2. On the contrary, PD2 shows obviously large photo current when the input is Y polarized light (Pol 11 in Fig. 4). However, if the input light is 45° linear polarized (Pol 6 in Fig. 4), both PDs measured the comparable photo currents. These reveal the capability of dual polarization detection.

### High speed measurement

**Figure 5:**
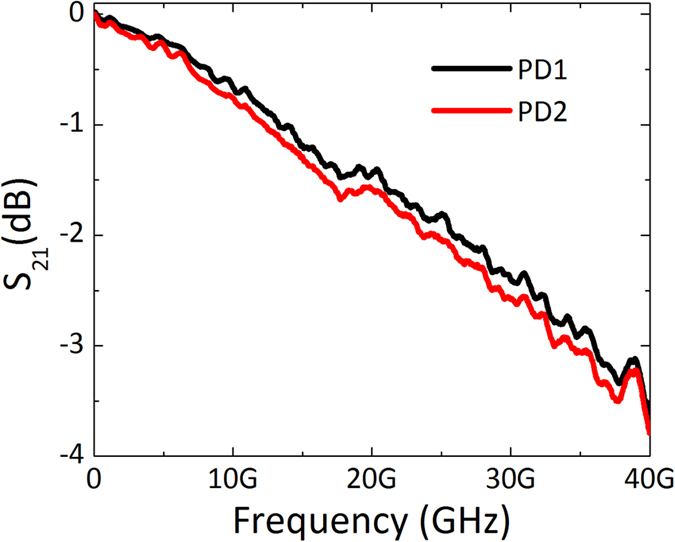
Bandwidth measurement results. The measured S21 for both the two Ge PDs when the reverse biased voltage is 3 V.

For characterizing the bandwidth, the frequency response of the receiver is measured based on the lightwave component analyzer (LCA). The modulated optical signal from the LCA at 1550 nm with power of about 5 dBm is vertically coupled into one of the Ge PDs. A polarization controller (PC) is used to maximize the coupling efficiency. Then the converted electrical signal is measured by the LCA through a RF cable. Meanwhile, the reverse biased voltage from the Keithley 2400 source meter is applied to the Ge PDs through a bias-tee. The measured S_21_ curves for the two PDs are shown in Fig. 5, and the bandwidths are about 36 GHz for both the two Ge PDs when the reverse biased voltage is 3 V.

**Figure 6:**
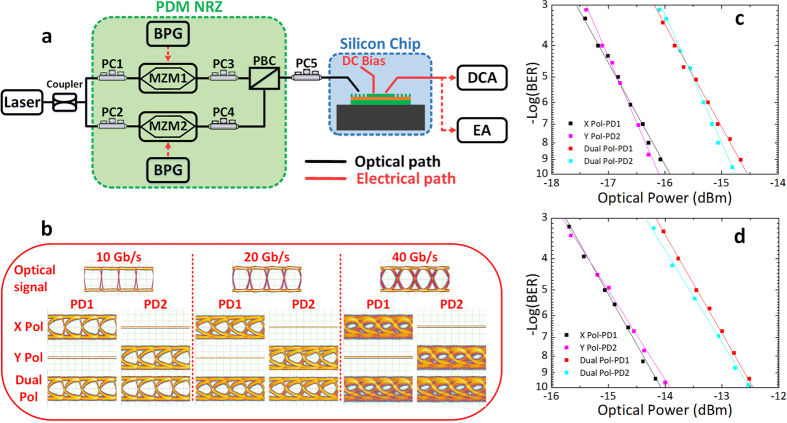
High speed data transmission experimental results. (**a**) Experimental setup. BPG: Bit pattern generator; MZM: Mach-Zehnder modulator; DCA: Digital communication analyzer; EA: Error analyzer. (**b**) The measured 10, 20 and 40 Gb/s eye diagrams by PD1 and PD2 for the X, Y and Dual polarized signals. (**c**) The measured 10 Gb/s and (**d**) 20 Gb/s BER as a function of received optical power from PD1 and PD2 when the input signals on both single and dual polarization cases.

The high speed experiment is performed with setup shown in Fig. 6(a). A CW light from a tunable laser is split and coupled into two modulation units (Mach-Zehnder modulators which were driven by the data streams at three different modulation rates with the pseudorandom bit steams (PRBS) length of 2^15^ − 1 from the bit pattern generator (BPG)) to obtain the two independent NRZ signals. Two polarization controllers (PC 1 and 2) are used to optimize the modulation performance. Assisting by another two PCs (PC 3 and 4), the modulated signals are combined by a polarization beam combiner (PBC), forming the PDM NRZ signals. Using the vertical fiber coupling system, the PDM signals are then coupled into the chip. The PC 5 is used to match the two orthogonal polarizations in the fiber and the waveguides modes in the device, respectively. The eye diagrams at both PDs are measured by the digital communication analyzer (DCA) and the results are shown in Fig. 6(b). In the measurement, one of the modulator can be disabled and the performances for single and dual polarization cases can be compared. For the X polarization state, only PD1 shows open eye diagram while PD2 shows nothing. On the contrary, PD2 shows the open eye diagram while PD1 shows nothing for Y polarization state. When the PDM signals with dual polarization states (X and Y polarization) are injected and matched to the 2D grating, both the two Ge PDs detected the same open eye diagrams. Therefore, the results validate sufficiently that the PDM-NRZ signals with two orthogonal polarization states can be detected by the proposed scheme. The conclusions are the same for all the 10, 20 and 40 Gb/s cases.

Finally, the BER measurements are performed for quantitatively characterizing the performance of the proposed device. For demonstration, only results at 10 and 20 Gb/s are measured. The 2D GC is designed to be balanced and both channels are equalized in the measurement. The recorded different BER as a function of received optical power are plotted in Fig. 6(c,d), for the cases of single (X or Y Polarization) and dual polarization (X and Y polarizations) states. The results accord well with the previous analysis and the power penalty for single and dual polarization is about 1.9 dB at 10^−9^ for both 10 and 20 Gb/s cases. The reasonable power penalty is caused by the polarization crosstalk of the input orthogonal optical signal.

It should be noted that the polarization scrambling will affect the experiment results, by disturbing the power coupled into each PD. In our experiments, the polarization controllers are optimized adaptively according to the measured photo currents and eye diagrams, and every effort had been done to minimize the measurement error. The polarization scrambling can be counteracted through adaptively controlling the polarization state of the input signals according to the signal quality after direct detection^30^, or using the polarization-maintaining fiber^31^.

## Conclusions

In conclusion, we have proposed and fabricated a silicon based dual-detector optical receiver, which can directly detect PDM signals. The proposed receiver consists of a polarization diversity GC and double sides illuminated Ge absorption region. A high responsivity of 1.06 A/W and a large bandwidth of 36 GHz are successfully achieved. The measured eye diagrams and BER results vindicate the capacity of demultiplexing and detecting PDM signals simultaneously. The proposed receiver with simple structure is cost-effective and can be potentially utilized in the short reach photonic link with direct detection.

## Methods

The Ge absorption rate vs. Ge length and the optical field distribution in the Ge region are simulated based on the FDTD Solutions. The proposed device is fabricated on the 220 nm thick SOI wafer with 2 μm buried oxide (BOX) at the Institute of Microelectronics (IME) in Singapore.

## Additional Information

**How to cite this article**: Chen, G. *et al*. A dual-detector optical receiver for PDM signals detection. *Sci. Rep*. **6**, 26469; doi: 10.1038/srep26469 (2016).

## References

[b1] Richardson D. J., Fini J. M. & Nelson L. E. Space-division multiplexing in optical fibres. Nature Photon. 7, 354–362 (2013).

[b2] Winzer P. J. Making spatial multiplexing a reality. Nature Photon. 8, 345–348 (2014).

[b3] Ding Y. . On-chip two-mode division multiplexing using tapered directional coupler-based mode multiplexer and demultiplexer. Opt. Express 21, 10376–10382 (2013). 23609748 10.1364/OE.21.010376

[b4] Dai D., Wang J. & Shi Y. Silicon mode (de)multiplexer enabling high capacity photonic networks-on-chip with a single-wavelength-carrier light. Opt. Lett. 38, 1422–1424 (2013). 23632505 10.1364/OL.38.001422

[b5] Ye M., Yu Y., Zou J., Yang W. & Zhang X. On-chip multiplexing conversion between wavelength division multiplexing–polarization division multiplexing and wavelength division multiplexing–mode division multiplexing. Opt. Lett. 39, 758–761 (2014). 24562199 10.1364/OL.39.000758

[b6] Wang J., Chen P., Chen S., Shi Y. & Dai D. Improved 8-channel silicon mode demultiplexer with grating polarizers. Opt. Express 22, 12799–12807 (2014). 24921475 10.1364/OE.22.012799

[b7] Luo L.-W. . WDM-compatible mode-division multiplexing on a silicon chip. Nature Commun. 5, 3069 (2014). 24423882 10.1038/ncomms4069

[b8] Stern B. . On-chip mode-division multiplexing switch. Optica 2, 530–535 (2015).

[b9] Ye M., Yu Y., Chen G., Luo Y. & Zhang X. On-chip WDM mode-division multiplexing interconnection with optional demodulation function. Opt. Express 23, 32130–32138 (2015). 26699003 10.1364/OE.23.032130

[b10] Evangelides S. G. Jr., Mollenauer L. F., Gordon J. P. & Bergano N. S. Polarization multiplexing with solitons. J. Lightw. Technol. 10, 28–35 (1992).

[b11] Michel J., Liu J. & Kimerling L. C. High-performance Ge-on-Si photodetector. Nature Photon. 4, 527–534 (2010).

[b12] Reed G. T., Mashanovich G., Gardes F. Y. & Thomson D. J. Silicon optical modulators. Nature Photon. 4, 518–526 (2010).

[b13] Hochberg M. & Baehr-Jones T. Towards fabless silicon photonics. Nature Photon. 4, 492–494 (2010).

[b14] Taillaert D. . A compact two-dimensional grating coupler used as a polarization splitter. Phot. Technol. Lett. 15, 1249–1251 (2003).

[b15] Bogaerts W. . A polarization-diversity wavelength duplexer circuit in silicon-on-insulator photonic wires. Opt. Express 15, 1567–1578 (2007). 19532389 10.1364/oe.15.001567

[b16] Fukuda H. . Silicon photonic circuit with polarization diversity. Opt. Express 16, 4872–4880 (2008). 18542586 10.1364/oe.16.004872

[b17] Doerr C. R. . Monolithic polarization and phase diversity coherent receiver in silicon. J. Lightw. Technol. 28, 520–525 (2010).

[b18] Doerr C. R. . Packaged monolithic silicon 112-Gb/s coherent receiver. Phot. Technol. Lett. 23, 762–764 (2011).

[b19] Xiang L. . SOI based ultracompact polarization insensitive filter for PDM signal processing. Opt. Lett. 38, 2379–2381 (2013). 23939054 10.1364/OL.38.002379

[b20] Dong P., Xie C. & Buhl L. L. Monolithic polarization diversity coherent receiver based on 120-degree optical hybrids on silicon. Opt. Express 22, 2119–2125 (2014). 24515221 10.1364/OE.22.002119

[b21] Dong P. . Monolithic silicon photonic integrated circuits for compact 100+Gb/s coherent optical receivers and transmitters. IEEE J. Sel. Top. Quantum Electron. 20, 150–157 (2014).

[b22] Ji H.-C., Lee J. H., Kim H., Park P. K. J. & Chung Y. C. Effect of PDL-induced coherent crosstalk on polarization-division-multiplexed direct-detection systems. Opt. Express 17, 1169–1177 (2009). 19188943 10.1364/oe.17.001169

[b23] Schmidt B. J., Lowery A. J. & Armstrong J. In Optical Fiber Communication Conference, Paper PDP18 (Anaheim, CA, USA, 2007).

[b24] Wang Z., Xie C. & Ren X. PMD and PDL impairments in polarization division multiplexing signals with direct detection. Opt. Express 17, 7993–8004 (2009). 19434131 10.1364/oe.17.007993

[b25] Al Amin A., Takahashi H., Morita I. & Tanaka H. 100-Gb/s direct-detection OFDM transmission on independent polarization tributaries. Phot. Technol. Lett. 22, 468–470 (2010).

[b26] Qian D., Cvijetic N., Hu J. & Wang T. 108 Gb/s OFDMA-PON with polarization multiplexing and direct detection. J. Lightw. Technol. 28, 484–493 (2010).

[b27] Shen Y. . Design of polarization de-multiplexer and PMD compensator for 112 Gb/s direct-detect PDM RZ-DQPSK systems. J. Lightw. Technol. 28, 3282–3293 (2010).

[b28] Peng W.-R., Morita I., Takahashi H. & Tsuritani T. Transmission of high-speed (>100 Gb/s) direct-detection optical OFDM superchannel. J. Lightw. Technol. 30, 2025–2034 (2012).

[b29] Morsy-Osman M., Chagnon M., Poulin M., Lessard S. & Plant D. V. 224-Gb/s 10-km transmission of PDM PAM-4 at 1.3 μm using a single intensity-modulated laser and a direct-detection MIMO DSP-based receiver. J. Lightw. Technol. 33, 1417–1424 (2015).

[b30] Cai J.-X. . In Optical Fiber Communication Conference, Paper PDP4 (San Diego, CA, USA, 2008).

[b31] Zhou X. . In Optical Fiber Communication Conference, Paper PDPB4 (San Diego, CA, USA, 2009).

